# Real-Time Label-Free Embolus Detection Using *In Vivo* Photoacoustic Flow Cytometry

**DOI:** 10.1371/journal.pone.0156269

**Published:** 2016-05-26

**Authors:** Mazen A. Juratli, Yulian A. Menyaev, Mustafa Sarimollaoglu, Eric R. Siegel, Dmitry A. Nedosekin, James Y. Suen, Alexander V. Melerzanov, Tareq A. Juratli, Ekaterina I. Galanzha, Vladimir P. Zharov

**Affiliations:** 1 Arkansas Nanomedicine Center, University of Arkansas for Medical Sciences (UAMS), Little Rock, AR, 72205, United States of America; 2 Department of General and Visceral Surgery, University Hospital Frankfurt, Goethe-University, Frankfurt am Main, Germany; 3 Department of Biostatistics, UAMS, Little Rock, AR, 72205, United States of America; 4 Department of Otolaryngology – Head and Neck Surgery, UAMS, Little Rock, AR, 72205, United States of America; 5 Moscow Institute of Physics and Technology (MIPT), Moscow Region 141700, Russia; 6 Department of Neurosurgery, University Hospital Dresden, Dresden, Germany; King Faisal Specialist Hospital & Research center, SAUDI ARABIA

## Abstract

Thromboembolic events are one of the world’s leading causes of death among patients. Embolus or clot formations have several etiologies including paraneoplastic, post-surgery, cauterization, transplantation, or extracorporeal circuits. Despite its medical significance, little progress has been made in early embolus detection, screening and control. The aim of our study is to test the utility of the *in vivo* photoacoustic (PA) flow cytometry (PAFC) technique for non-invasive embolus detection in real-time. Using *in vivo* PAFC, emboli were non-invasively monitored in the bloodstream of two different mouse models. The tumor-free mouse model consisted of two groups, one in which the limbs were clamped to produce vessel stasis (7 procedures), and one where the mice underwent surgery (7 procedures). The melanoma-bearing mouse model also consisted of two groups, one in which the implanted tumor underwent compression (8 procedures), and one where a surgical excision of the implanted tumor was performed (8 procedures). We demonstrated that the PAFC can detect a single embolus, and has the ability to distinguish between erythrocyte–rich (red) and leukocyte/platelet-rich (white) emboli in small vessels. We show that, in tumor-bearing mice, the level of circulating emboli was increased compared to tumor-free mice (*p* = 0.0013). The number of circulating emboli temporarily increased in the tumor-free control mice during vessel stasis (*p* = 0.033) and after surgical excisions (signed-rank *p* = 0.031). Similar observations were noted during tumor compression (*p* = 0.013) and after tumor excisions (*p* = 0.012). For the first time, it was possible to detect unlabeled emboli *in vivo* non-invasively, and to confirm the presence of pigmented tumor cells within circulating emboli. The insight on embolus dynamics during cancer progression and medical procedures highlight the clinical potential of PAFC for early detection of cancer and surgery-induced emboli to prevent the fatal thromboembolic complications by well-timed therapy.

## Introduction

When a blood vessel is injured, the body’s normal physiological response is to form thrombi to prevent blood loss [1–3]. Alternatively, even in the absence of injury, many medical procedures such as surgery, cauterization, transplantation, and extracorporeal circuits, amplified by high blood pressure, atherosclerosis, and other risk factors, may provoke formation of thrombi and lead to pulmonary embolism, stroke, heart attack, and other cardiovascular disorders, which remain among the world’s leading causes of death [4]. An explosion of data in the past few years has underscored the fact that thrombotic events are the second leading cause of death among cancer patients [5–7]. Embolism, which is a complication of thrombus formation, can be induced by surgery, transplantation, isolated limb perfusion and implantation of central or venous catheters. Pathophysiology traditionally emphasizes the factors that constitute the Virchow triad of blood stasis, vessel wall changes, and hypercoagulability. In addition, inflammation can also contribute to a hypercoagulable state and endothelial damage [8–10]. Finally, embolism was shown to be one of the factors promoting metastasis by forming platelet-rich aggregates around circulating tumor cells (CTCs), thereby protecting the CTCs in blood flow against shear forces or immune responses [11–13]. Thus, on one hand cancer can accelerate thrombosis, while on the other thrombosis can enhance metastasis in a complex, bidirectional relationship called the platelet–cancer-loop [14].

Despite the clear medical significance, little progress has been made in developing highly sensitive methods for embolus detection. Commonly used *ex vivo* methods have low sensitivity and low diagnostic value [15]. Doppler ultrasound techniques have shown promise for detecting a large thrombus *in vivo*, but this method cannot detect micro-thrombi, suffers from artifacts, and requires highly trained personnel [16]. As a result, many thromboses remain undetectable, unless they result in clinical phenomena [2,17]. About 5–10% of patients die because of the failure to diagnose rather than inadequate therapy [18,19]. Although the risk of recurrence decreases with longer durations of preventive anticoagulant treatment, there is no tool for estimating the risk of thrombus-related complications versus the risk of bleeding-associated complications, particularly hemorrhage [20].

As an alternative, photoacoustic (PA) imaging techniques have shown the potential to detect adherent thrombi and detect emboli non-invasively in deep tissues [21,22]. PA method is unique in its ability to perform high-resolution analysis of light-absorbing targets in deep tissues. Multiple published papers demonstrate that the penetration depth for PA technique in diagnostics can reach several cm [23]. Another example is detection of tiny blood vessels at 3–9 mm depth using NIR light [24].

However, application of the PA technique to the real-time monitoring of embolus dynamics during a medical intervention in large vessels is challenging. The main goal in this paper is to demonstrate that the *in vivo* PAFC can rapidly detect emboli triggered by melanoma and various medical procedures based on detection of transient changes in blood absorption caused by presence of circulating emboli. Early embolus detection followed by well-timed anticoagulant therapy could open the way to preventing lethal complications from thrombi that are impossible to detect with existing techniques.

## Materials and Methods

### Photoacoustic flow cytometry (PAFC)

**Fig 1:**
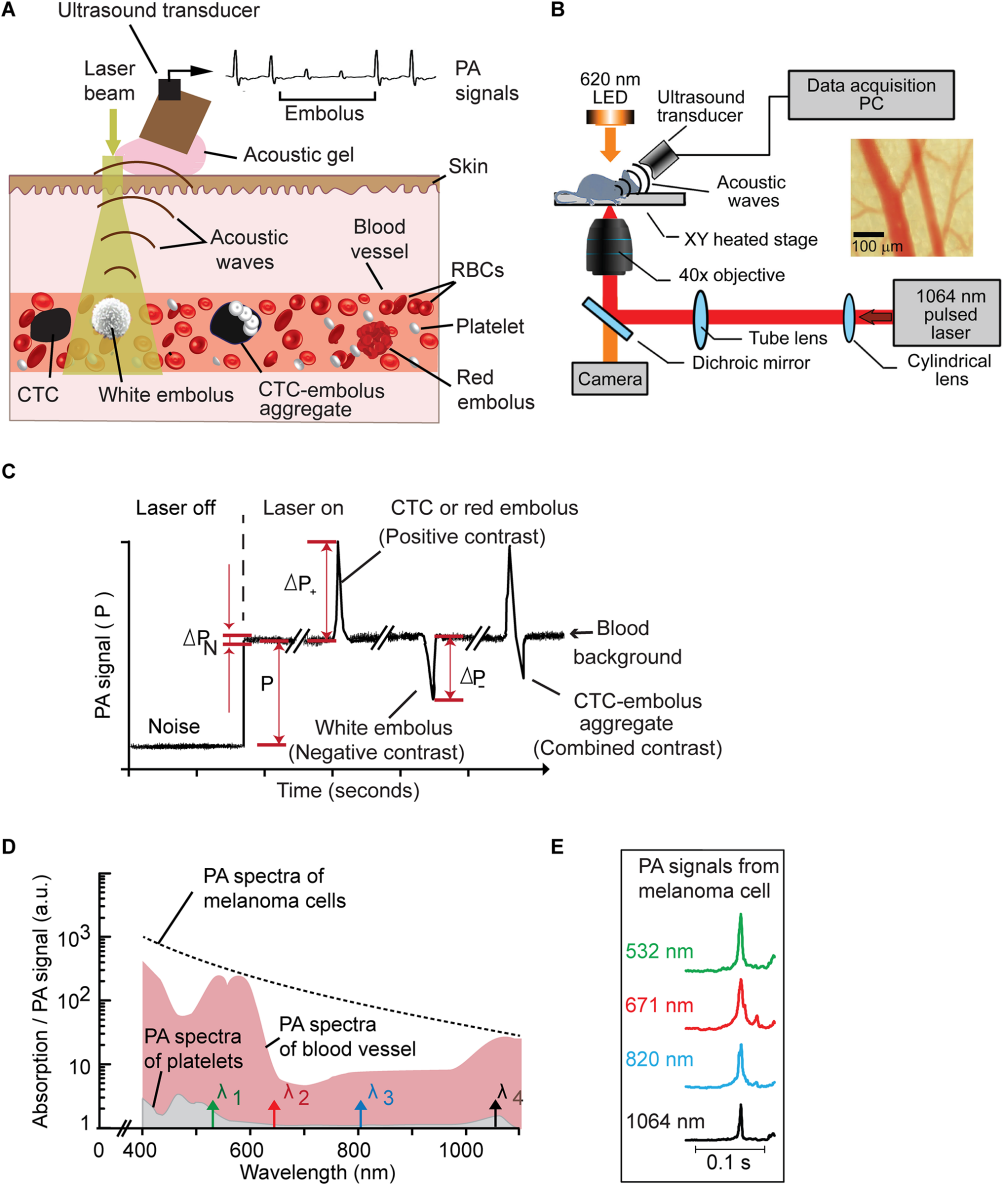
Principles of *in vivo* photoacoustic (PA) flow cytometry (PAFC). (A) Principle of *in vivo* PAFC setup using high pulse repetition rate (PRR) laser and broadband ultrasound transducer to detect abnormal cells in superficial vessels. (B) Schematics of the *in vivo* PAFC. (C) PA trace showing signals having positive (CTC or red embolus), negative (white embolus) and combined (CTC-embolus aggregate) contrast. (D) Absorption and PA spectra of 50-μm-diameter veins in mouse ear (red region), platelets (gray region) and B16F10 melanoma cells (dashed curve). (E) Typical views of PA signals from B16F10 melanoma cells at different laser wavelengths.

PAFC platform was built upon the Eclipse E400 microscope (Nikon Instruments, Inc.) and equipped with a 1064 nm pulsed fiber-based laser (model MOPA-M-1-10-1, Multiwave Photonics S.A., Portugal) (Fig 1A and 1B). The laser was set to operate at a 10 kHz pulse repetition rate, 10 ns pulse width, and 10 μJ pulse energy. Laser beam was focused into vessels using a 40x micro-objective (NA 0.65, model PlanFluor, Nikon Instruments Inc.). Laser energy was controlled using a PM100USB power and energy meter and an S314C sensor (both from Thorlabs, Inc.). An LED light source and a CCD camera (model Xli DX-2M, Brunel Microscopes Ltd, UK) were used to visualize the tissue and align the laser beam onto a vessel. Data acquisition was triggered by a photodetector (150 MHz, model PDA10A, Thorlabs Inc.). Laser-induced acoustic waves were detected by an ultrasound transducer (2.25 MHz bandwidth, model V-323-SM, Olympus NTD, Waltham, MA) and further amplified by a preamplifier (50 kHz– 5 MHz bandwidth, 54 dB gain, model 5662, Panametrics NDT). Standard ultrasound gel (Aquasonic Clear, Parker Labs Inc.) was placed on the skin for acoustic coupling.

PA signals from the ultrasound transducer were acquired by a digitizer module (14 bit resolution, 125 MHz sampling frequency, model AD484, 4DSP Inc., Reno, NV). Data acquisition and post-processing operations were implemented in MATLAB (MathWorks, Natick, MA) and performed on a workstation (Precision T7500, Dell, Round Rock, TX). Acquired signals were first averaged 10 times to increase the signal to noise ratio, then peak to peak amplitude of PA waveforms were traced. In this signal trace, relatively stable PA signals from a blood vessel constitute a flat line (Fig 1C); a particle with higher absorbance than blood (e.g., CTC) produces a sharp positive peak. The appearance of a negative peak indicates the passage of a low absorbing object (e.g., white embolus) (Fig 1D). Signal traces were filtered using a 10 Hz high-pass filter to eliminate low frequency artifacts related to movement of blood vessel caused by heartbeats or breathing. Traces were then analyzed for the presence of positive and negative PA peaks exceeding the thresholds obtained from a control experiment.

### Principle of label-free PA detection of emboli *in vivo*

The principle of label-free PA *in vivo* embolus detection was described in our previous work [25]. Briefly, the presence of many erythrocytes consisting of strongly absorbing hemoglobin in the detection volume creates a constant PA background (Fig 1C). A transient increase in blood absorbance may be caused by presence of an erythrocyte-rich (red) dense blood embolus or pigmented melanoma CTC resulting in a positive PA peak (Fig 1C, left). Conversely, a leukocyte and/or platelet-rich (white) blood embolus having a lower absorbance in the laser beam than that of blood causing a transient decrease in PA signal amplitude, which produce a negative peak (dip) on a PA trace (Fig 1C, middle). If an embolus is formed by a mixture of red–white emboli or contains a pigmented CTC then a distinctive pattern of transient positive and negative PA signals (Fig 1C, right) may be seen on the PA trace. Further analysis of embolus content may be done using multispectral detection with laser pulses of different wavelengths probing sample absorption (Fig 1D and 1E). The PAFC platform presented here was equipped with four laser sources (532, 671, 820, and 1,064 nm) allowing multicolor characterization of circulating objects (Fig 1E). However, in current work we focused on a single color detection using only 1064 nm laser with lower attenuation and scattering in tissues. Single laser detection allowed identification of white/red emboli, melanoma CTC (much higher positive single amplitude compared to emboli) and mixed CTC/white emboli aggregates.

### Mouse model

All protocols of animal-related experiments were approved by the University of Arkansas for Medical Sciences, Institutional Animal Care and Use Committee. The *in vivo* capabilities of the PAFC were tested in blood microvessels (diameter: 30–70 μm) in the thin ear tissue (depth: ~250 μm) of nude (nu/nu) mice aged 6–8 weeks and weighing 20–22 g. All procedures were performed completely under anesthesia (~1.5–2.0% Isoflorane). After the procedure, the mice were euthanized via CO_2_ asphyxiation.

For *in vivo* monitoring of emboli, mice were anesthetized by isoflurane inhalation and placed on a temperature-controlled microscope stage (37°C). The intact right ear of each mouse was spread over the stage glass window, allowing continuous monitoring of emboli in the ear vein with our *in vivo* PAFC setup. *In vivo* mouse-model experiments involved both tumor-free mice as controls and melanoma-bearing mice inoculated with cells of the B16F10 cell line to provide the tumors.

#### (A) Tumor-free mouse model

**Fig 2:**
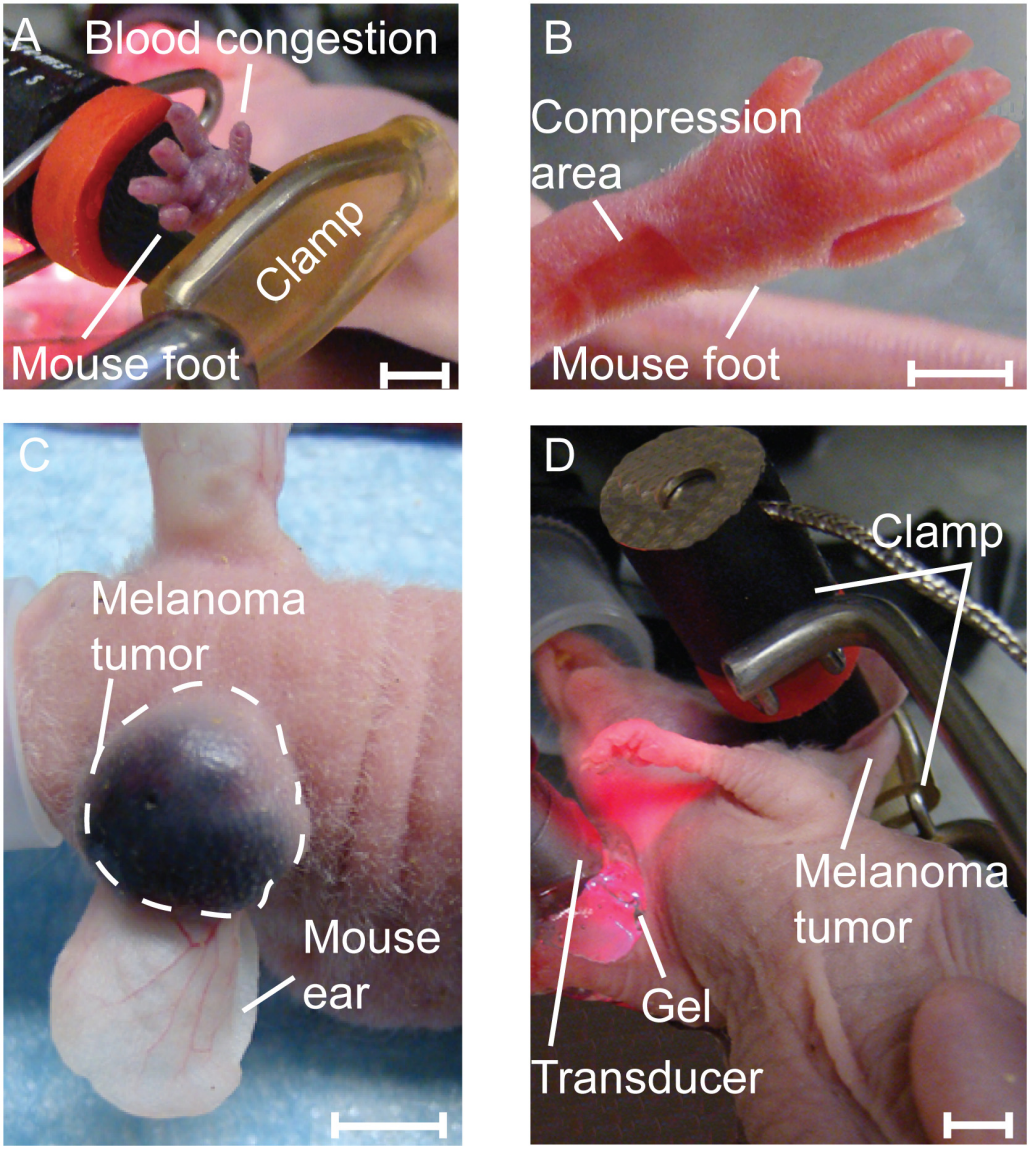
Images of healthy and melanoma-bearing mice during intervention. (A) Blood congestion in healthy mouse foot during clamping. (B) Healthy mouse foot after clamp removal with no bleeding or tissue damage. (C) Melanoma tumor in the mouse ear before applying the 120 g pressure. (D) *In vivo* real-time PA detection of emboli during ear B16F10-tumor compression (120 g weight) in nude mouse ear. Scale bars are 0.5 cm.

To study the effect of tissue compression on embolus dynamics, a clamp with 400 g of pressure and no sharp edges was applied to the legs of mice (7 procedures) for 30 minutes with the use of digital pressure-controller software (DI-100; Loadstar Sensors). The contact area was approximately 0.5 cm^2^ (Fig 2A and 2B). Continuous monitoring for emboli was maintained throughout the 30 minutes during compression and continued for another 160 minutes after the compression.

In the second mouse group (7 procedures), blood-vessel endothelial damage was induced by surgery to study the effect on the production of emboli. In every case, the back of each mouse was washed with chlorhexidine and air-dried, and then an excision was made by cutting through the skin and muscle on the mouse back. An ultrasound transducer was placed on an ear vein. Each excision took approximately 3 minutes. Monitoring was maintained throughout the surgery and for 190 minutes afterward.

#### (B) Melanoma-bearing mouse model

When tumor volumes in melanoma-bearing mice reached 600 mm^3^, the mice (8 procedures) were placed under the microscope and continuously monitored with our *in vivo* PAFC setup. To approximate the pressure of palpation during the examination of the tumor, pressure was applied to the tumor for 30 minutes with a 120-g weight using digital pressure-controller software (Loadstar Sensors, DI-100). The area of contact between the weight and the tumor was typically 0.5 cm^2^. (Fig 2C and 2D) Continuous monitoring was initiated 30 minutes before compression began, maintained during all 30 minutes of compression, and continued for another 160 minutes after the compression ended.

To study the effect of surgical tumor excision on embolus dynamics, melanoma-bearing mice (8 procedures) were placed under the microscope when the tumor volume reached 600 mm^3^. PAFC was used to monitor circulating white emboli for 30 minutes before a biopsy incision was made in the tumor on the mouse ear. Each biopsy took approximately one minute to perform. Continuous monitoring was maintained throughout the biopsy period and for 190 minutes afterward. Each procedure was applied once per mouse for all experiments.

### Statistical analyses

SAS version 9.4 (The SAS Institute, Cary, NC) was employed for all statistical analysis. All hypothesis tests were two-sided and employed a 5%-alpha significance level, except where indicated below. Detection rates were compared between time periods via mixed-models Poisson regression with unstructured autocovariance matrix, except for the vessel-damage experiment, which employed one-sided signed-rank tests at alpha = 0.05 because of the complete absence of embolus counts in all mice before surgery. To assess the difference in embolus-detection rates between melanoma-bearing and non-tumor mice, the monitoring times before the mouse’s first manipulation was used. Accordingly, embolus-detection rates in each mouse were calculated as a weighted average by taking the sum of all embolus counts divided by the sum of all pre-manipulation monitoring times. The resulting normalized detection rates were compared between groups via Wilcoxon rank-sum test. To assess embolus dynamics over time after B16F10 inoculation, we used Poisson regression with generalized estimating equations to accommodate the longitudinal nature of the data.

## Results

### Correlation between emboli and melanoma before intervention

**Fig 3:**
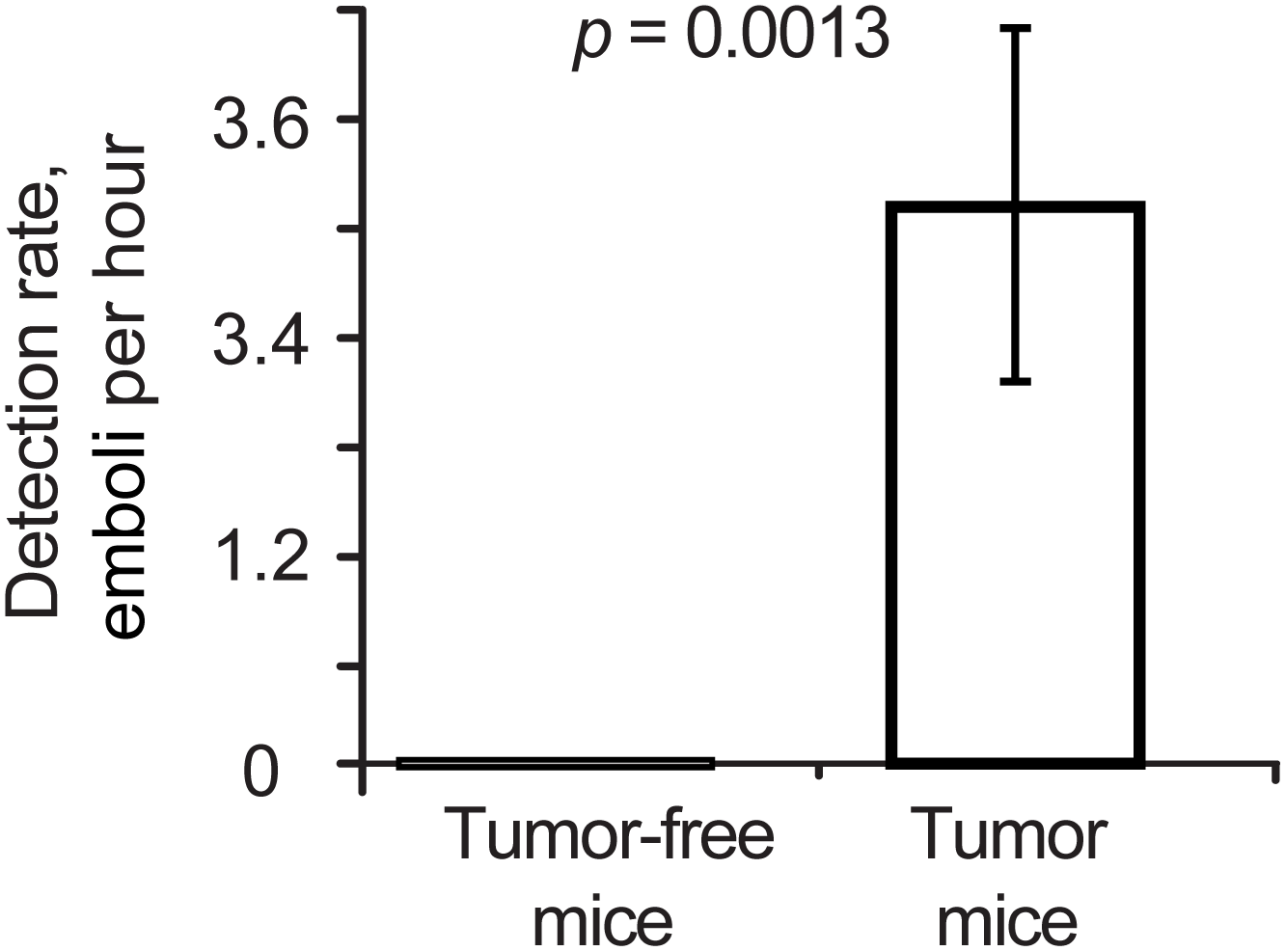
Relationship between embolus and melanoma. Detection rate of white emboli in 16 melanoma-bearing mice and 14 tumor-free mice (*p* = 0.0013). Values and error bars represent the mean and the standard error of the mean (SEM) of embolus counts.

To study the relationship between melanoma and emboli presence, we quantified the number of circulating white emboli in 14 tumor-free control mice and in 16 melanoma-bearing mice. In each mouse, an ear vein (diameter: 40–60 μm; depth: ~150 μm) was monitored for approximately 30 minutes, when the tumor volume reached 600 mm^3^. No white emboli were detected by the PA probe among the tumor-free control mice, during 570 cumulative total minutes of monitoring, whereas a total of ~50 white emboli were detected among the melanoma-bearing mice during 930 cumulative total minutes of monitoring. The mean and SEM of the normalized detection rate in the melanoma-bearing mice was 0.51 ± 0.18 emboli/10min, which represented a significant increase in comparison with the tumor-free control mice (*p* = 0.0013). Thus, the presence of circulating white emboli as quantified by PAFC was positively correlated with the presence of a malignant tumor in our mouse models (Fig 3).

### Emboli dynamics after interventions in tumor-free control mice

Tissue compression and surgery may cause formation of emboli. To prove this hypothesis we enumerated emboli in tumor-free mice during these interventions.

**Fig 4:**
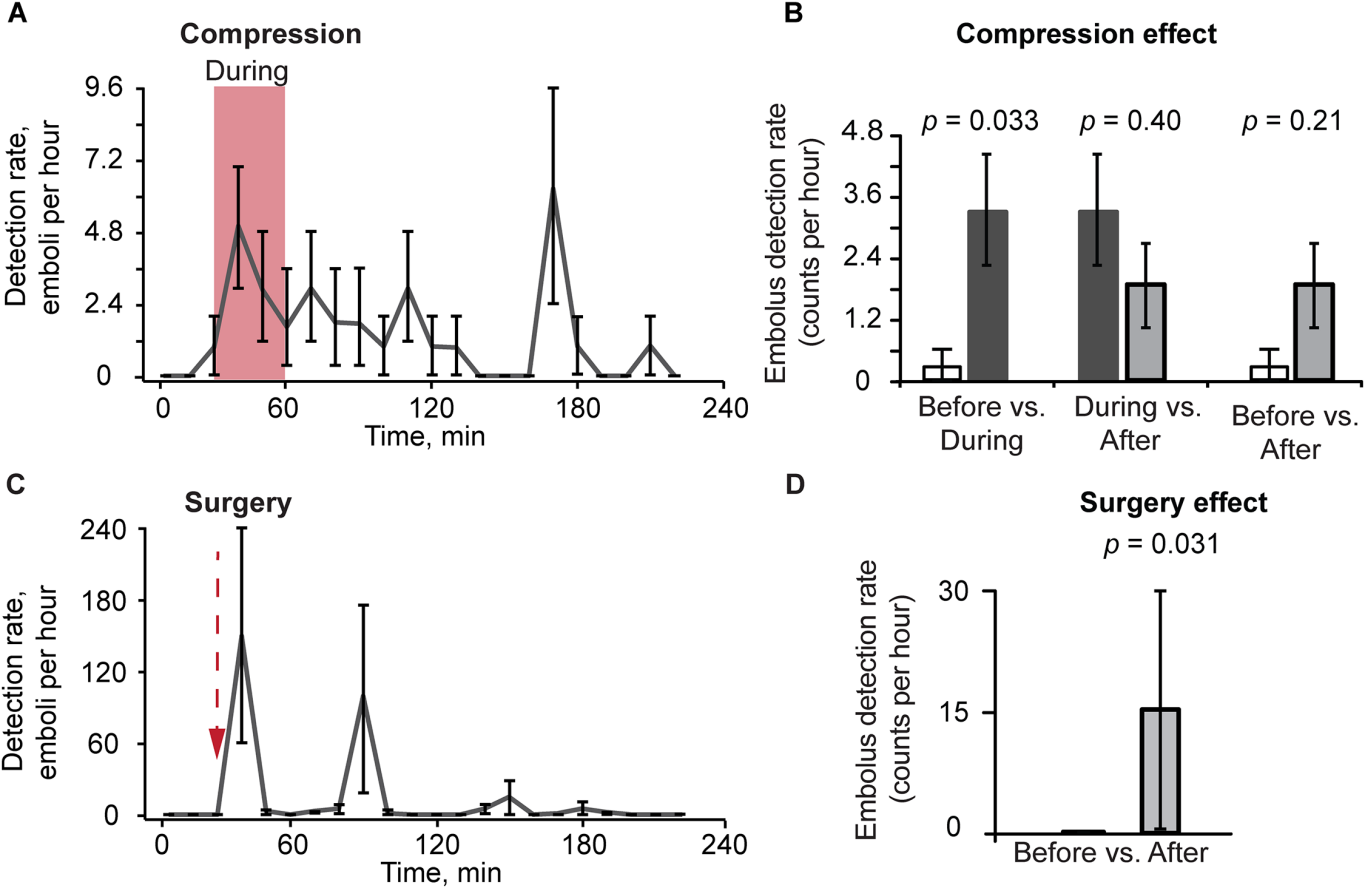
Embolus dynamics in control mice before, during and after compression and surgery. (A) Profile plot of average detection rate (emboli per hour) from before, 30 minutes during (red region) and after compression with a clamp (400 g) (7 procedures). Detection-rate values and error bars represent the mean and SEM of detection rates during successive 10-minute time intervals, and are positioned at the intervals’ midpoints. (B) Effects of compression effect on embolus detection rates. Values (error bars) represent the estimates (90% confidence intervals) of detection rates (emboli per hour) before compression vs. during and after compression and during vs. after compression as determined from mixed-models Poisson-regression analysis (7 procedures). (C) Profile plot of average detection rates (emboli per hour) for 30 minutes before and 190 minutes after surgery. Values and error bars represent the mean and SEM of detection rates during successive 10 minute time intervals (N7 procedures). Red arrow indicates initiation of the surgery (duration ~3 min). (D) Embolus detection rates in counts per hour before vs. after surgery. Values (error bars) represent estimated detection rates (SEM) (7 procedures).

First, we performed emboli enumeration during limb compression on tumor-free mice (7 procedures). Before the intervention an average detection rate was 0.03 emboli/10min (only red emboli were detected, no white emboli). The number of red emboli in ear vessels of tumor-free mice increased within 5 minutes of flow restriction, and reached an average of 3.31 emboli/hr during the compression (10-fold increase compared to the period before the compression, *p* = 0.033). The highest detection rate for emboli was observed ~100 minutes after removal of the compression (Fig 4A). Further, the detection rate decreased to an average of 1.85 emboli/hr (*p* = 0.21) during the 160 minutes after the compression ended (Fig 4A and 4B).

Surgical excision was performed on tumor-free mice (7 procedures). Immediately after cutting through skin and muscle, red emboli were detected at an average rate ±SEM of 9.17±7.93 counts/hr with a median (quartiles) of 1.86 (0.00–2.85) counts/hr (1-sided signed-rank *p* = 0.062). Forty minutes later, white emboli began to appear. Their overall detection rate had an average ±SEM of 6.70±6.52 counts/hr with a median (quartiles) of 0.00 (0.00–0.52) counts/hr (one-sided signed-rank *p* = 0.12). After the complete surgical excision was made, the detection rate of both colors of emboli had an average ± SEM of 15.9 ± 14.4 counts/hr with a median (quartiles) of 1.86 (0.33–3.04) counts/hr (1-sided signed-rank *p* = 0.031) (Fig 4C and 4D). The red-embolus count immediately increased after the surgical excision. The first increase in emboli appeared 3–13 minutes after surgical excision, but the maximum rate was detected 10–60 minutes after the beginning of the surgery. On the other side, the number of white emboli started to increase 50 minutes after surgery began, while the red emboli disappeared. No emboli were detected in these mice before the intervention.

### Embolus dynamics after medical interventions in melanoma-bearing mice

**Fig 5:**
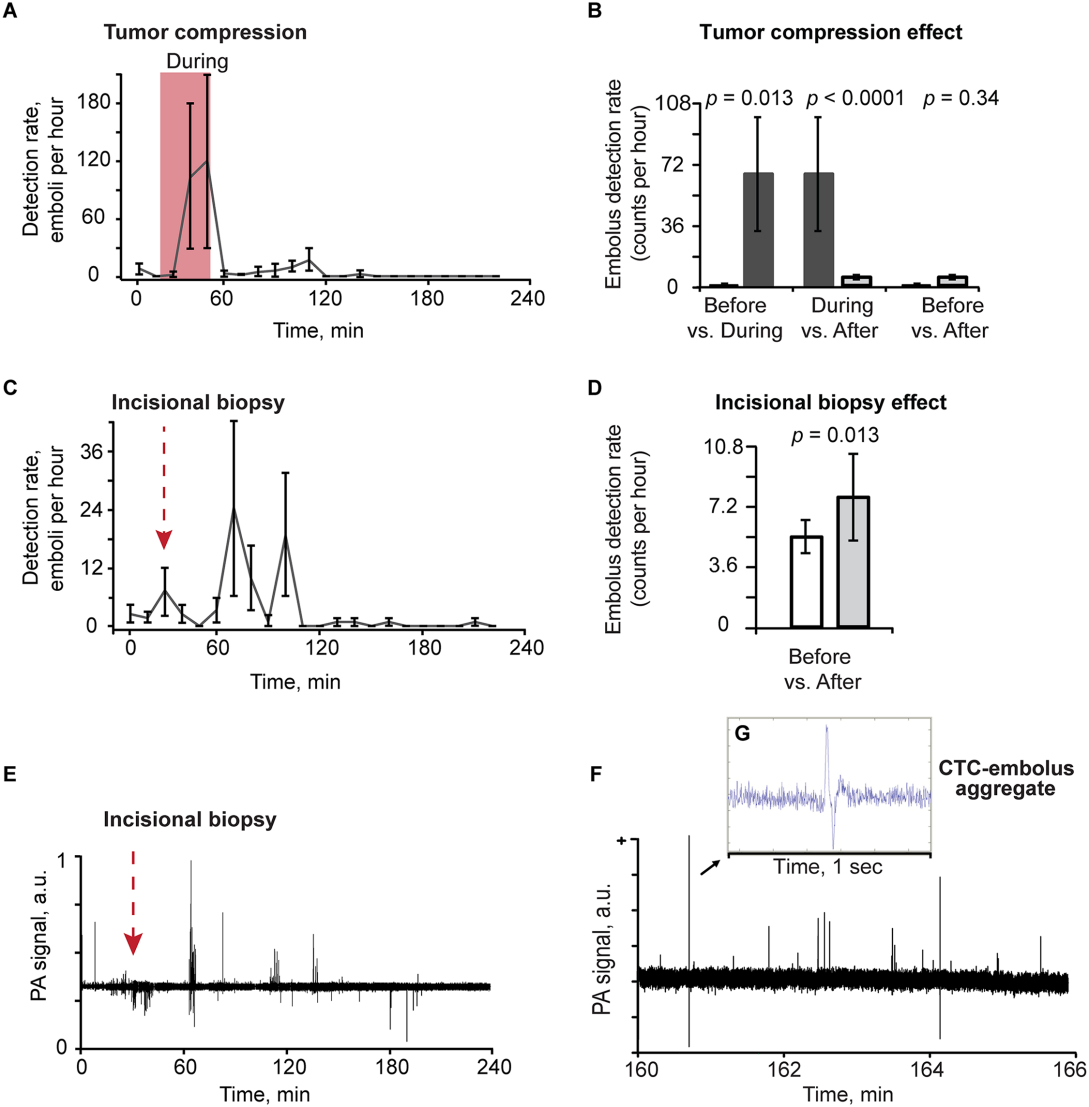
Embolus dynamics in tumor-bearing mice before, during and after compression and biopsy. (A) Profile plot of average detection rate (emboli per hour) from before, during (red region) and after tumor compression with a clamp (8 procedures). Detection-rate values and error bars represent the mean and SEM of detection rates during successive 10-minute time intervals, and are positioned at the intervals’ midpoints. (B) Effects of compression on embolus-detection rates. (C) Profile plot of average embolus-detection rate (counts per hour) for before, during and after a biopsy incision. Red arrow indicates initiation of the procedure (8 procedures). (D) Embolus-detection rates in counts per hour before vs. after a surgical tumor excision. (E) Sample trace of PA signals before and after a representative biopsy. Red arrow indicates initiation of the biopsy procedure. (F) Sample PA signal trace with detection of embolus in the blood vessel of a melanoma-bearing mouse after application of a 120-g weight on the tumor. (G) Shows details of combined PA contrast. (A,C) Values and error bars represent the mean and SEM of embolus detection rates during each 10-minute bin. (B,D) Values (error bars) represent the estimates (90% confidence intervals) determined from mixed-models Poisson-regression analysis.

To study the effect of tumor compression on embolus dynamics, eight melanoma-bearing mice were used. Before compression, the average white-embolus rate was 2.48 emboli/hr. During compression with a 120 g weight on the leg of a mouse, the white-embolus detection rate increased to 65.3 emboli/hr (*p* = 0.013) and with the cessation of compression decreased to 6.77 emboli/hr (*p* = 0.34). The rate of white emboli was 9.6 times higher during compression than after the end of compression (*p*< 0.0001) (Fig 5A and 5B). The white-embolus rate increased immediately upon tumor manipulation, with two maximum rates occurring bimodally at 20 minutes during compression and at 50 minutes after removal of compression.

Embolus dynamics were also studied in an additional group of eight melanoma-bearing mice who underwent a surgical tumor excision (8 procedures) using a scalpel. The white-embolus detection rate was 5.19 emboli/hr before the procedure started. During and after the incision, the white-embolus detection rate increased to 7.59 emboli/hr (*p* = 0.012) (Fig 5C, 5D and 5E). The first increase in the white-embolus rate appeared 1 to 2 minutes after the procedure, and the maximum rate occurred during the procedure and up to 90 minutes afterward.

After the tumor compression and biopsy in four melanoma-bearing mice, which were used above, we observed a total of seven signals that had a combined positive and negative PA contrast. An example of these signals which associated with aggregate of CTC and white embolus is presented in Fig 5G.

## Discussion

Thrombotic events are the second leading cause of death among cancer patients. Surgery, transplantation, and extracorporeal circuits may contribute to the formation of emboli, which leads to increased morbidity and mortality. Despite its medical significance, little progress has been made in early embolus diagnosis, prevention, treatment, and control. In particular, the incidence of complicating strokes during coronary artery bypass grafting reaches 6–9% [26,27].

The focus of these preclinical studies was proof of concept of the applicability of the PAFC as an advanced research tool in animal models. We found that compression on tumor-free mouse legs led to a significant (16-fold) formation of emboli associated with endothelial damage and denudation. In this experiment we observed a decreased blood circulation in the lower limb vessels in the area under the clamp, which may cause embolus formation during the compression period.

Similar-appearing embolus dynamics were observed during surgery in tumor-free control mice. The difference in the number of emboli may be related to the type of tissue compressed or the number of vessels damaged, suggesting that embolus formation is a universal phenomenon in response to mechanical injury. Our studies of the effects of tumor manipulations on the number of emboli showed a 10-fold increase in their number after tumor compression with a 120-g weight (around 5 times the mouse weight) and a 7-fold increase after surgical tumor excision.

It has been reported previously that compression and/or surgery are associated with an increased risk of thromboembolic events [8–10]. The pathophysiology traditionally emphasizes the series of factors that constitute the Virchow triad of blood stasis, changes in the vessel wall, and hypercoagulability. Inflammation can also play a role by additionally influencing hypercoagulability and endothelial damage. The inflammatory response after compression or surgery, is initiated by a cytokine "storm" and occurs within hours after manipulation. It creates a prothrombotic environment that is further accentuated by several cellular processes including neutrophil extracellular traps formation, platelet activation, and the generation of tissue factor-bearing microparticles.

The close relationship between cancer and thrombosis has been described in 1865 from Armand Trousseau. Cancer and thrombosis are linked by several pathophysiological mechanisms [5,7], including 1) enhanced blood coagulation through thrombin and fibrin formation and through platelet activation by the release of procoagulants from tumor cells and indirectly through the activation of endothelial cells and leukocytes [28]; 2) radiation- and drug-induced coagulation [29–31]; and 3) tumor-caused mechanical vascular stasis [5,12]. As a result, cancer can trigger thrombosis, and conversely, thrombotic phenomena can lead to metastasis progression [6,7,32]. However, no technique has been available to explore and understand these phenomena in order to develop strategies for the treatment and prevention of emboli

In the melanoma-bearing mouse model, we have observed a correlation between the presence of white emboli and melanoma. Pilot studies suggest that elevated numbers of white emboli can be an independent marker of adverse prognosis of thrombosis in patients with cancer [33]. This has been demonstrated in several malignancies, including cervical, lung and gastric cancers [5,33]; but there was no method available to rapidly control emboli. In current studies, we found that the embolus dynamics were positively correlated with the presence of a malignant tumor in our mouse models. We also detected increased counts of CTC–emboli aggregates in blood vessels after tumor manipulations. Thus, *in vivo* detection of these aggregates has shed light on the role of platelets in promoting CTC migration to distant organs: covered with a coat of platelets, CTCs acquire the ability to evade the body’s immune system [14,34]. However, to determine the link between CTCs and emboli, and the roles of platelets and leukocytes in the early stage of tumor formation, additional studies are required.

Our work shows that the PAFC can fill the gap in a completely unexplored area of embolization research related to the detection of previously uncontrollable embolus dynamics during medical interventions and cancer progression.

Previously, we and others have explored optical resolution of *in vivo* PAFC, in which the resolution is determined by the optical parameters; in particular, the minimum width of a focused linear laser beam. Due to strong light scattering in tissue, high spatial resolution at the level of 5–10 μm has been achieved in 30–50-μm-diameter superficial vessels at a shallow depth of 0.1–0.2 mm [35–40], Indeed, the clinical potential and safety of PA devices have also been demonstrated in many pilot studies in humans, including the monitoring of cerebral blood oxygenation in approximately 1-cm-diameter human jugular veins at a depth of 1–2 cm [41] and the imaging of vessels at a depth of up to 5–7 cm [42]. However, application of the PA technique to embolus detection has not previously been attempted. The additional advantage of the PAFC is its ability to spectrally identify red and white emboli in a label-free manner, thereby allowing us to exclude the possible influence of the labeling procedure on embolus detection.

Clinical perspectives of PAFC system depend on its ability to monitor deep large vessels to improve the rate of detection for rare circulating objects and/or monitor anatomically important vessels. Photoacoustic detection provides a unique possibility to monitor deep vessels through detection of ultrasound waves originating in deep vessels. In this application detection resolution is determined by acoustic resolution and can be dramatically improved through the use of high frequency focused broadband transducers. We used a 1064 nm laser allowing deep penetration in tissues and still providing good PA contrast of hemoglobin at this wavelength (Fig 1D). We have also developed PAFC with the optical clearing that allows additionally increase PA contrast of deep vessels [38]. PA assessment of the carotid artery is also possible [22] and may provide an opportunity for early stroke prevention using PAFC technology.

The major challenge in translation of the PAFC system into clinics would be in the optimization of light delivery to the deep tissues. Multiple solutions exist for various anatomical structures including recycling of scattered photons [43] and light delivery via the pharynx [22]. Thus, depending on the site of monitoring (for example neck vs. arm vessels) various light delivering strategies should be applied to maximize light delivery without a risk of laser damage to the tissues.

## Conclusion

Using PAFC to study the size, rate, and composition (e.g., red or white) of emboli could provide insights into their role in the development of deadly complications associated with the failure of early embolus diagnosis rather than inadequate therapy. The high sensitivity of PAFC enables detecting the earliest appearance of emboli in the circulation. We expect that the PAFC platform, attached to a patient’s hand or to bypass tubes in the operating room or during a transfusion procedure, will provide well-timed warnings of dangerous embolus formation. Moreover, this instrument could be useful as a means to directly examine and quantify the antithrombotic activity of new pharmaceuticals and to understand the etiology and pathogenesis of thromboembolism.
